# The angiogenic gene profile of circulating endothelial progenitor cells from ischemic stroke patients

**DOI:** 10.1186/2045-824X-5-3

**Published:** 2013-02-06

**Authors:** Míriam Navarro-Sobrino, Mar Hernández-Guillamon, Israel Fernandez-Cadenas, Marc Ribó, Ignacio A Romero, Pierre-Olivier Couraud, Babette Barbash Weksler, Joan Montaner, Anna Rosell

**Affiliations:** 1 Neurovascular Research Laboratory and Neurovascular Unit. Neurology and Medicine Department, Universitat Autònoma de Barcelona. Vall d’Hebron Research Institute of Vall d’Hebron Hospital, Pg Vall d’Hebron 119-129, Barcelona, 08035, Spain; 2 Department of Life Sciences, The Open University, Milton Keynes, UK; 3 Division of Hematology and Medical Oncology, Institut Cochin, Université Paris Descartes, Paris, France; 4 Weill Medical College of Cornell University, New York, NY, USA

**Keywords:** Ischemic stroke, Angiogenesis, Endothelial progenitor cells, Gene expression, Vasculogenesis

## Abstract

**Background:**

The identification of circulating endothelial progenitor cells (EPCs) has introduced new possibilities for cell-based treatments for stroke. We tested the angiogenic gene expression of outgrowth endothelial cells (OECs), an EPC subtype capable to shape vessel structures.

**Methods:**

OECs (at colony or mature stages) from ischemic stroke patients (n=8) were characterized using the RT^2^ Profiler^TM^ human angiogenesis PCR Array, and human microvascular endothelial cells (hCMEC/D3) were used as an expression reference of endothelial cells.

**Results:**

Colony-OECs showed higher expression of *CCL2*, *ID3*, *IGF-1*, *MMP9*, *TGFBR1*, *TNFAIP2*, *TNF* and *TGFB1*. However, *BAI-1*, *NRP2*, *THBS1*, *MMP2* and *VEGFC* expression was increased in mature-OECs (p<0.05). ID3 (p=0.008) and TGFBR1 (p=0.03) genes remained significantly overexpressed in colony-OECs compared to mature-OECs or hCMEC/D3. MMP9 levels were significantly increased in colony-OECs (p=0.025) compared to mature-OECs. Moreover, MMP-2, VEGF-C, THBS1 and NRP-2 gene expression was also significantly increased in mature-OECs compared to hCMEC/D3 (p<0.05). Some of these genes were positively validated by RT-PCR.

**Conclusion:**

Our study shows that OECs from stroke patients present higher levels of pro-angiogenic factors at early stages, decreasing in mature OECs when they become more similar to mature microvascular endothelial cells.

## Introduction

Endothelial progenitor cells (EPCs) is a minor population of circulating mononuclear cells that participates in adult neovascularization in pathological and physiological processes. Current research on EPCs in adults holds great promise and is receiving much attention due to their contribution to neoangiogenesis in vascular injuries, such as wound healing, limb ischemia, myocardial infarction, atherosclerosis or stroke [1].

An approach to isolate EPCs from peripheral blood utilizes *in vitro* culture and produces two distinct EPC subtypes which have been named circulating angiogenic cells or “early EPCs” (eEPCs) and “outgrowth endothelial cells” (OECs). OECs are also known as endothelial colony-forming cells (ECFCs) or late EPCs because of their late appearance in culture and their ability to grow from expanding colonies [2]. OECs have been directly involved in vascular repair by forming perfused human neo-vessels when injected subcutaneously into immune deficient mice [3]. It has already been demonstrated that OECs are more efficiently isolated from ischemic stroke patients than control subjects together with their ability to shape capillary-like structures *in vitro*[4]. For the specific purpose of vascular disease modelling, OECs should be the preferred EPC subtype to use, and a better understanding of their angiogenic characteristics in their different stages could be very useful to assess their regenerative potential in cell based therapies for ischemic stroke patients. However, the molecular features that define OECs differentiation in ischemic stroke patients are still unknown.

To better understand the molecular program of OECs in ischemic stroke, our aim was to examine the angiogenesis-related gene expression profile of OECs from ischemic stroke patients at initial colony (colony-OECs) or mature stages (mature-OECs), and compared them to mature human cerebral microvascular endothelial cells (hCMEC/D3) as a reference of endothelial cell expression.

## Materials and methods

### Ethics statement

The study was approved by the Ethics Committee of our institution and conducted in accordance with the Declaration of Helsinki. All patients or relatives and healthy controls gave written informed consent.

### Isolation and culture EPCs

**Figure 1:**
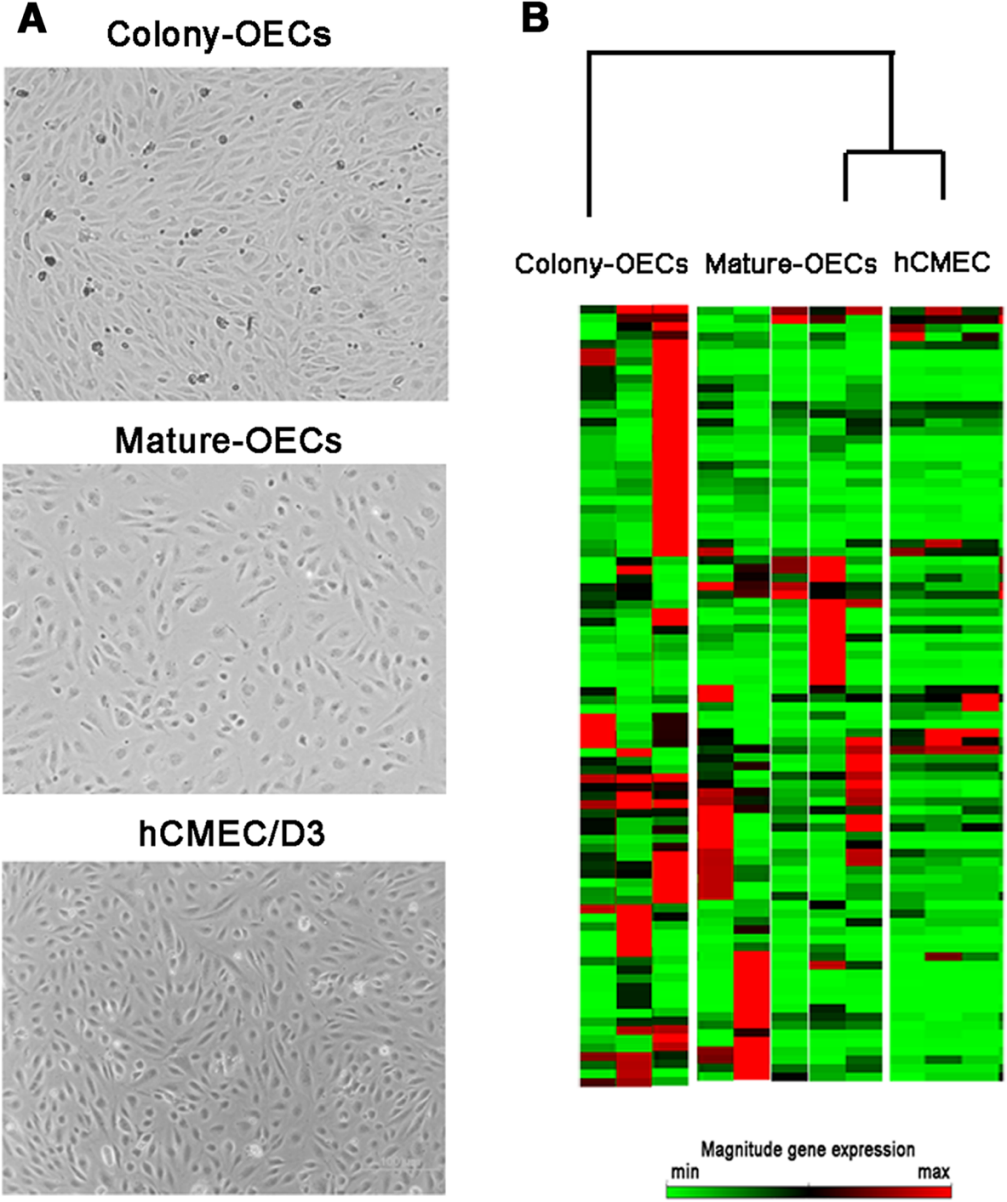
**Representative cell culture images and gene expression profile of the studied cells. A**) Representative phase contrast images of colony-OECs (40× magnification) and mature-OECs (100x magnification) from ischemic stroke patients and hCMEC/D3 (100× magnification). **B**) Heat maps illustrating gene-expression profile of patients included in colony-OECs, mature-OECs and hCMEC/D3 groups. The green color indicates low expression and the red color indicates high expression.

Peripheral blood (20 ml) was obtained in EDTA-anticoagulated tubes from patients who had suffered a non-lacunar ischemic stroke (n=8) involving the middle cerebral artery (MCA) territory between 24 h and 7 days after onset of symptoms, and who had been admitted to the emergency department of our center. OECs were isolated from peripheral blood as previously described [4]. Colony-OECs appeared as rounded expanding colonies, while later acquisition of confluent cobblestone-shaped in monolayers was identified as mature-OECs (Figure 1A). hCMEC/D3 cells, which are derived by immortalization of human brain primary microvascular endothelial cells were grown as previously described [5]. Additionally, EPCs from sex- and age-matched control subjects free of ischemic events, inflammatory or infectious diseases (n=17) were initially cultured but cell cultures did not yield OEC cells.

A detailed history of any concomitant medication was obtained from each patient and all were scored for stroke severity and neurological status on admission and on follow-up visits according to the National Institutes of Health Stroke Scale (NIHSS). Functional outcome was defined by modified Rankin Scale (mRS) at three months and patients with mRS >2 points were considered functionally dependent.

### RNA isolation and quality control

RNA was obtained from cell cultures for both early-colony stage OECs and mature OECs. Since those cells obtained from the colony stage were not further expanded, different patients were included in the mature-OECs group. The RNA from the mature-OECs was isolated from cells between the 4^th^ and 10^th^ passage since after 12 population doublings mature-OECs started to change their morphology probably undergoing to senescence. The colony-OECs were isolated before the first passage was done. Finally, hCMEC/D3 cells line was included as a reference of human endothelial cell type.

Colony-OECs obtained from ischemic stroke patients (n=3) were isolated using a cell scraper. Mature-OECs (1×10^5^ cells/mL) taken between passages 4 and 10 from ischemic stroke patients (n=5) and hCMEC/D3 (1×10^5^ cells/mL) at different passages (from 32 to 34; n=3) were seeded in twelve-well plates with EGM-2 (Clonetics®, CA, USA) and maintained until confluence reached about 90%. A total of 11 samples were included in the study. Total RNA was extracted with the RNeasy Mini kit (Qiagen, CA, USA). The RNA concentration and quality were measured using the Bioanalyzer 2100 system (Agilent, CA, USA). All samples presented high-quality RNA with RNA integrity numbers above 8.5. cDNA was pre-amplified using RT^2^ Nano PreAmp cDNA Synthesis Primer Mix Human Angiogenesis kits according to manufacturer’s instructions (SabBioscience, Qiagen). RT^2^ Profiler human angiogenesis PCR array was performed for quantitative PCR in the ABI 7000 system (Applied Biosystems, USA) with the following cycling conditions; 10 min at 95°C, 15 s at 95°C, 1 min 60°C for 40 cycles with a final infinite 4°C hold.

### Data normalization

Five endogenous control genes *glucuronidase β* (*GUS β*), *hypoxanthine guanine* (*HPRT1*), *heat-shock protein 90* (*HSP90*), *glyceraldehyde phosphate dehidrogenase* (*GAPDH*), and *βactin* (*ACT β*)] present on the RT^2^ Profiler human angiogenesis PCR Array were used for data normalization. Each replicate cycle threshold (Ct) was normalized to the geometric median Ct of 5 endogenous controls per plate. Ct was defined as 35 for the ΔCt calculation when the signal was under detectable limits. The relative amount of transcripts in the colony-OECs and mature-OECs samples was calculated compared to our expression reference in hCMEC/D3. Results were calculated using the 2^-ΔΔCt^ method [6]. Changes in gene expression between colony-OECs, mature-OECs and hCMEC/D3 cells are shown as a fold increase or decrease. Heat maps (color-coded graphs with groups in columns and genes in rows) were generated by using the web based program of RT^2^ profiler PCR Array Data Analysis.

### Real time PCR analysis

Quantitative Real-time PCR (qRT-PCR) was used to measure mRNA expression levels of *MMP2*, *MMP9, VEGF-C, NRP-2 and THBS1* as a validation of the array data. Those probes were available in our laboratory, therefore the selection of genes for validation was random and not dependant on the significance level or the fold change. The same samples (n=11) used in the RT^2^ Profiler human angiogenesis PCR Array were included. The mRNA levels were quantified using TaqMan Hs00234422_m1 MMP2 (for *MMP2*), Hs00234579_m1 MMP9 (for *MMP9*), VEGFC Hs00153458_m1 VEGFC (for VEGF-C), Hs00962908_m1 THBS1 (for THBS1), Hs00187290_m1 NRP2 (for NRP-2) and Hs00181777_m1 BAI1 (for BAI1). The expression of the housekeeping gene peptidylprolylisomerase A (*PPIA*; probe Hs99999904_m1) was quantified as a reference to normalize all values. Real-time PCRs were run in triplicate and analyzed using the Applied Biosystems SDS 7500 system software (Applied Biosystems). The results are expressed as percentages based on a calibration sample used in all experiments.

### Statistical analysis

The statistical significance was set at p value less than 0.05 and a mean difference equal to or greater than 2-fold change in expression levels. Normal distribution of the variables was tested using the Shapiro-Wilk test. Differences between colony-OECs, mature-OECs and hCMEC/D3 groups were determined using the One-Way ANOVA or Kruskal Wallis and Mann Whitney tests for normal and non-normal distributions, respectively. Data were expressed as mean fold change ± SD for normal distributed variables or median (interquartile range) for non-normal distributed variables. All statistical analyses were performed with SPSS version 15.0 software. To account for multiple statistical testing, false discovery rate (FDR) *q*-values were calculated.

## Results

The stroke patients included in the study were under different secondary prevention treatments. One patient in each study-group was receiving oral anticoagulation therapy, 2 patients in colony-OECs and 3 in mature-OECs group were under antiplatelet agents. In addition, 2 patients in colony-OECs group and 3 in mature-OECs group were under statins. Moreover, a patient in the colony-OECs group showed a previous stroke.

Baseline NIHSS was similar in patients from colony-OECs and mature-OECs groups (14 ± 7.2 vs. 8.2 ± 5.8; p=0.297 respectively) and also NIHSS at discharge (12.3 ± 6.5 vs. 11.6 ± 15.9; p=0.513 respectively). Regarding functional outcome no differences were found between patients from colony-OECs and mature-OECs groups (4 ± 2 vs. 2.4 ± 2.2; p=0.263 respectively). None of the analyzed genes showed any correlation between their expression level and the clinical outcome. White blood cell counts of the stroke patients included in the study were not statistically different (p=0.65).

The Human Angiogenesis RT^2^ Profiler PCR Array profiles the expression of 84 key genes involved in the biological processes of angiogenesis. Over 90% of the transcripts (n= 75) were detected whereas the expression of *ANGPT1*, *CXCL9*, *IL8*, *COL18A1*, *COL4A3*, *CCL11, HAND2*, *IFN-G* and *TIMP-3* was undetected by this technique. Quality control parameters included in the Human Angiogenesis RT2 Profiler PCR Array (positive PCR controls and reverse transcription controls) showed a good reproducibility and efficiency based on the web based program of RT2 profiler PCR Array Data Analysis.

Overall, the analysis of the heat maps showed that mature-OECs gene-expression pattern partially matched hCMEC/D3 (Figure 1B). Our results showed that in the colony-OECs group, 45 genes were overexpressed whereas only 13 genes were underexpressed. Similarly, the mature-OECs group showed 31 genes overexpressed and 15 underexpressed compared to hCMEC/D3 cells. At the same time, we only identified 13 genes displaying a significantly different expression between colony-OECs and mature-OECs and all of them were overexpressed in OECs compared to hCMEC/D3 as shown in Table 1.

**Table 1:** Identification of differentially expressed transcripts between the Colony- or Mature-OECs compared to hCMEC/D3 cells

**Symbol**	**Gene name**	**Colony-OECs fold change (n=3)**	**Mature-OECs fold change (n=5)**	**p value**	***q*value**
**ID3**	Inhibitor of DNA binding 3	4.5 ± 0.4 *#	2.1 ± 1.4	0.008	0.750
**TNFAIP2**	Tumor necrosis factor alpha-induced protein 2	47.2 (13-455) #	3.9 (1-9)	0.018	0.750
**TGFβR1**	Transforming growth factor beta receptor 1	3.5 (3-28) #	1.8 (0.7-2)	0.030	0.750
**TGFβ1**	Transforming growth factor beta 1	2.2 ± 0.2 *	1.4 ± 0.7	0.036	0.750
**CCL2**	Chemokine (C-C motif) ligand 2	48.7 (28-117) *#	12.8 (0.7-20)	0.036	0.500
**THBS1**	Thrombospondin 1	22.7 (19-23)	5.8 (3-30) *	0.037	0.375
**BAI 1**	Brain-specific angiogenesis inhibitor 1	43 (12-103)	14.2 (2-33) *	0.037	0.600
**MMP2**	Matrix metallopeptidase 2	508.7 (306-600) *	67.2 (32-595) *	0.042	0.333
**NRP2**	Neuropilin 2	76.9 (22-185)	12.7 (9-238) *	0.043	0.300
**TNF**	Tumor necrosis factor	320.4 (39-531) *	0.9 (0.5-9)	0.046	0.313
**IGF1**	Insulin-like growth factor 1	11393.9 (1682-13738) *#	1.4 (0.8-8)	0.046	0.341
**VEGFC**	Vascular endothelial growth factor C	5.7 (2-6)	5 (3-9) *	0.048	0.268
**MMP9**	Matrix metalloprotease 9	2125.8 (627-2563)*#	0.6 (0.1-2)	0.049	0.250

Data are expressed as mean fold change ± SD or median (interquartile range) as appropriate.

* p<0.05 and fold change ≥2 Colony-OECs or Mature-OECs vs hCMEC/D3 cells; # p<0.05 and fold change ≥2 Colony-OECs vs Mature-OECs.

*CCL2* showed an increase in colony-OECs compare to mature-OECs or hCMEC/D3 cells [fold change 48.7 (28–117); p=0.036]*. ID3* was also increased in colony-OECs when compared to others groups [fold change 4.5 ± 0.4; p=0.008]. Moreover*, IGF-1* and *MMP9* were also overexpressed in colony-OECs compared to both mature-OECs and hCMEC/D3 [fold change 11393.9 (1682–13738) and 2125.8 (627–2563), respectively] as shown in Table 1. *TNF* and *TGFβ1* showed higher gene expression [fold change 320.4 (39–531) and 2.2 ± 0.2, respectively] in colony-OECs compared to hCMEC/D3. Interestingly, *TGFβR1* and *TNFAIP2* also showed an increase [fold change 3.5 (3–28) and 47.2 (13–455), respectively] only in colony-OECs compared to mature-OECs.

On the other hand, we observed a significant increase in *BAI-*1 in mature-OECs compared to hCMEC/D3 [fold change 14.2 (2–33); p=0.037]. Similarly, our results showed that *NRP2, THBS1* and *VEGFC* were increased in mature-OECs when compared to hCMEC/D3 [fold change 12.7 (9–238); 5.8 (3–9) and 5 (3–9), respectively] as shown in Table 1. Finally, only the expression of *MMP2* was overexpressed in both colony-OECs and mature-OECs compared to hCMEC/D3 [fold change 508.7 (306–600) and 67.2 (32–595), respectively]. After performing Bonferroni post-test analysis for multiple comparisons in normal-distributed genes, only *ID3* and *TGFβR1* genes remained significantly overexpressed in colony-OECs compared to mature-OECs or hCMEC/D3. Finally, when applying a correction for multiple testing on all distributed genes analyzed, none of the *q* values obtained was statistically significant (Table 1).

**Figure 2:**
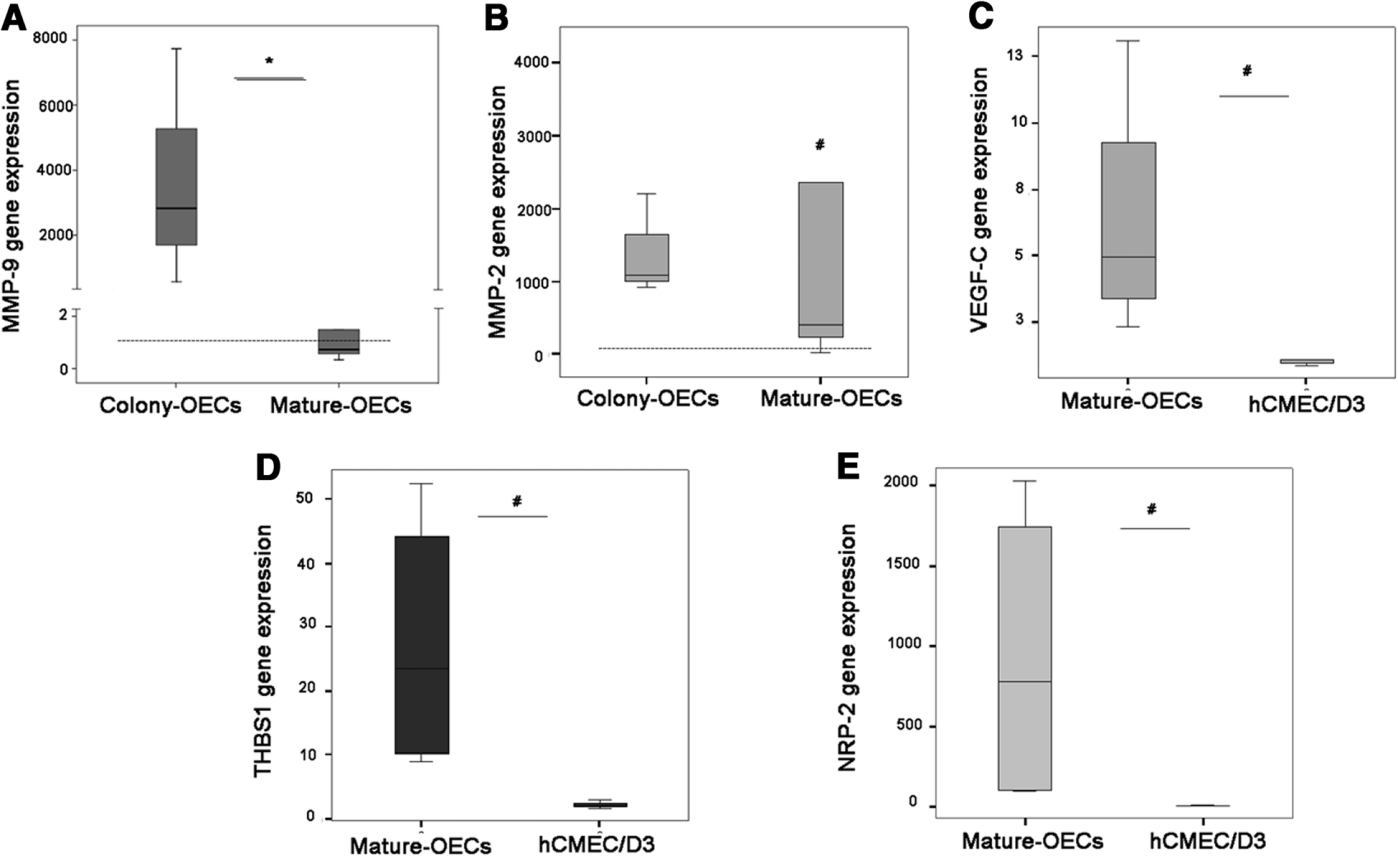
**Validation of *MMP2, MMP9, VEGF-C, THBS1 and NRP2 *mRNA levels by qRT-PCR. A**) *MMP9* mRNA levels in colony-OECs (n=3) and mature-OECs (n=5). **B**) *MMP2* mRNA levels in colony-OECs (n=3) and mature-OECs (n=5). **C**) VEGF-C gene expression levels in mature-OECs (n=4) and hCMEC/D3 (n=3). **D**) THBS1 mRNA levels in mature-OECs (n=4) and hCMEC/D3 (n=3). **E**) NRP-2 mRNA in mature-OECs (n=4) and hCMEC/D3 (n=3). Expression level is relative to calibrator sample and dashed line indicates median expression for hCMEC/D3 reference. * p<0.05 (colony vs. mature-OECs); # p<0.05 (vs. hCMEC/D3).

To validate our results by another technique MMP2, MMP9, VEGF-C, THBS1 and NRP-2 mRNA expression was measured using qRT-PCR. Figure 2A shows that *MMP9* levels were significantly increased in colony-OECs (p=0.025) compared to mature-OECs and also overexpressed compared to hCMEC/D3 (p=0.05). In colony-OECs we also observed a trend to increased *MMP2* levels compared to hCMEC/D3 (p=0.05) and a significant overexpression in mature-OECs compared to hCMEC/D3 (p=0.025); see Figure 2B. Moreover, VEGF-C, THBS1 and NRP-2 gene expression was also significantly increased (p=0.034) in mature-OECs compared to hCMEC/D3 as shown in Figure 2.

This data confirms the results obtained in the RT2 profiler PCR Array and validates the analysis.

## Discussion

This study reveals for the first time the angiogenic gene-expression profile of OECs from ischemic stroke patients. Our results show that early colony-OECs from ischemic stroke patients display higher expression of proangiogenic-related genes, while expression of these set of genes in more mature populations (mature-OECs) is more similar to human cerebral microvascular endothelial cells.

A recent study has revealed changes in gene expression of late outgrowth EPC-derived endothelial cells from systemic sclerosis patients that could contribute to the endothelial dysfunction and may be relevant to the development of the vasculopathy [7]. However, it is completely unknown the gene expression profile of EPCs from ischemic stroke patients.

Other authors have studied the differentiation process of cord blood-derived EPCs showing that the first stage involves the expression of genes related to cell adhesion to extracellular matrix; during the second stage, gene-expression profile reveals transcription of cell cycle and antiapoptotic genes; finally, after the proliferative stage, adherent EPCs acquire additional endothelial-specific characteristics through the expression of endothelial markers [8]. Our results in the present study show that some of the genes previously described as expressed in EPCs from cord blood or peripheral blood of healthy volunteers such as *IGF-1*, *MMP9*, *MMP2* and *ID3*[8,9] are also expressed in EPCs from ischemic stroke patients. Additionally, it is known that *IGF-1* is highly expressed in EPCs compared to mature endothelial cells or monocytes enhancing EPC differentiation involving PI3-kinase/phosphorylated Akt pathways of cell survival and proliferation [10,11]. Our data also confirms that *IGF-1* mRNA level is also increased in colony-OECs derived from stroke patients, suggesting a role for *IGF-1* during the differentiation process of these cells.

Furthermore, it is well established that secreted matrix metalloproteinases (MMPs) from EPCs have a pivotal role in the ischemia-induced neovascularization since they actively participate in matrix degradation [12]. In this context, we show that *MMP9* and *MMP2* are highly expressed in colony-OECs, further reinforcing the functional properties of this population. We also successfully validated the *MMP2*, *MMP9, VEGF-C, THBS1* and *NRP-2* results by qRT-PCR showing that the RT2 profiler PCR Array is a good method to assess gene expression levels even when differences in expression are small.

In addition, our findings also prove the overexpression of *ID3* and *TGFβR1* genes in colony-OECs, suggesting a role in the proliferation and differentiation of colony-OECs to endothelial cells. In fact, previous reports have demonstrated that *ID3* −/− mutant mice show a markedly impaired EPC mobilization [9] and *TGFβR1* has also been involved in the mobilization and differentiation of cord blood-derived EPCs [13]. Recently, the capacity of EPCs to support the activity and function of resident differentiated cells by paracrine mechanisms has focused increased attention [14]. Our study also demonstrates that colony-OECs show a higher proangiogenic gene expression pattern, suggesting that colony-OECs could potentially present a higher secretion of angiogenic growth factors.

The current study has revealed that the gene expression profile of mature-OECs from ischemic stroke patients partially resembles that of hCMEC/D3 cells. Recently, it has been reported that OECs acquire additional endothelial-specific characteristics based on transcriptomic-, proteomic-, and ultrastructural- analysis [15]. Consistent with that study, our results also demonstrate that mature-OECs show an increase in *MMP2*, *THBS1* and *VEGF-C*, reinforcing the endothelial nature of mature-OECs. Interestingly, we also observe an increase in mature-OECs expression of *BAI-1*, a member of the secretin receptor family involved in the inhibition of angiogenesis, supporting the hypothesis that angiogenesis is controlled by a local balance between stimulators and inhibitors.

In the context of cerebral ischemia, the first pre-clinical studies testing the therapeutic potential of EPCs administrated early EPCs [16,17] but lately, new studies are exploring the role of OECs [18]. It is important to define the nature of these cells in culture to identify the best population for transplantation.

A recent publication demonstrated that time in culture conditions did not alter phenotype of OECs with no significant change in antigen expression between early (passage 0–2), mid (passage 4–6) and late passage (passage 6–9) cells. Furthermore, they confirmed that late outgrowth endotelial progenitor cells resembled mature human umbilical vein endothelial cells (HUVEC) [19].

In conclusion, the present study shows that the expression pattern of the angiogenic-related genes from OECs of ischemic patents changes during their expansion but preserves the pro-angiogenic potential despite the ischemic insult. Therefore, it would be of great value to determine in which differentiation stage (i.e., whether colony or mature) would OECs be more effective in cell-based therapies, making them an attractive tool to be tested for autologous transplantation after ischemic stroke.

This study certainly includes some limitations. We included stroke patients between 24 h and 7 days after onset of symptoms because we demonstrated in a previous study that OECs from subacute strokes (obtained after 24 h of the symptoms onset) showed a higher expression and secretion of angiogenic factors than OECs obtained in the first hours following stroke [4]. However, the temporal profile of OECs maturation in terms of angiogenic factors gene expression needs to be confirmed at different time points in a larger cohort of stroke patients. We focused our study in ischemic stroke patients because cell cultures from sex- and age-matched controls did not yield OECs under the same cell culture conditions. In this regard, we have already demonstrated that circulating EPCs are reduced in control subjects compared to stroke patients [4]. Moreover, other authors have shown that hormonal status influences circulating EPC levels being significantly reduced in postmenopausal women related to reduce estradiol levels [20]. Since our control group was primarily composed by women in menopausal age (76.5%, 70.6±7.4 years old) this could also partially explain our difficulty. Finally, it would be interesting to compare gene expression patterns of OECs with those of primary endothelial cells. However, the advantage of using hCMEC/D3 cells is that they model the human blood brain barrier (BBB) and avoid the inherent variability as well as the difficulty of sourcing that apply to primary endothelial cultures. Further studies, must confirmed our results in a larger number of patients and further explored at functional level to define its biological implications.

## Competing interest

The authors declare that they have no competing interests.

## Authors’ contributions

M N-S carried out the array experiments, statistical analysis and wrote the paper. AR guided the research, analyzed the results and critiqued each draft of the manuscript. M H-G participated in the design of the study and helped to draft and review the manuscript. MR participated in the collection of the blood samples. I F-C performed the RT-PCR study. J M designed and helped to draft the manuscript. IR, P-OC and BBW are contributing authors since they provided the hCMEC/D3 cells under a Material Transfer Agreement. All authors read and approved the final version of the manuscript.
